# Secretin Modulates the Postnatal Development of Mouse Cerebellar Cortex Via PKA- and ERK-dependent Pathways

**DOI:** 10.3389/fncel.2017.00382

**Published:** 2017-11-30

**Authors:** Lei Wang, Li Zhang, Billy K. C. Chow

**Affiliations:** ^1^School of Biological Sciences, University of Hong Kong, Pokfulam, Hong Kong; ^2^GHM Institute of CNS Regeneration, Jinan University, Guangzhou, China

**Keywords:** secretin, cerebellar development, Purkinje cell morphogenesis, granular cell migration, cell apoptosis, PKA, ERK1/2

## Abstract

Postnatal development of the cerebellum is critical for its intact function such as motor coordination and has been implicated in the pathogenesis of psychiatric disorders. We previously reported that deprivation of secretin (SCT) from cerebellar Purkinje neurons impaired motor coordination and motor learning function, while leaving the potential role of SCT in cerebellar development to be determined. SCT and its receptor (SCTR) were constitutively expressed in the postnatal cerebellum in a temporal and cell-specific manner. Using a SCT knockout mouse model, we provided direct evidence showing altered developmental patterns of Purkinje cells (PCs) and granular cells (GCs). SCT deprivation reduced the PC density, impaired the PC dendritic formation, induced accelerated GC migration and potentiated cerebellar apoptosis. Furthermore, our results indicated the involvement of protein kinase A (PKA) and extracellular signal regulated kinase (ERK) signaling pathways in SCT-mediated protective effects against neuronal apoptosis. Results of this study illustrated a novel function of SCT in the postnatal development of cerebellum, emphasizing the necessary role of SCT in cerebellar-related functions.

## Introduction

The cerebellum has been well-established as the control center for motor coordination and motor learning in mammalian species while there is a growing acceptance for its implication in the cognitive function (Wagner et al., 2017). The highly foliated and stratified structure of cerebellar cortex is formed through a continuous process beginning at embryonic day (E) 11.5 in rodents (Hibi and Shimizu, 2012). During postnatal morphogenesis and development, the cerebellum undergoes dramatic changes as neural progenitor cells undergo mitosis, cell fate specification, radial migration, neurite growth and spine formation. Each of these cellular events is governed by both intrinsic determinants and environmental stimuli (reviewed in Leto et al., 2016). In particular, the maturation of Purkinje cells (PCs) and the proliferation and migration of granular cells (GCs) are two critical and interacting events among these developmental progresses (reviewed in Marzban et al., 2014). GCs, the most abundant neurons in central nervous system, undergo intensive proliferation within the external granular layer (EGL), followed by inward radial migration and maturation during the first two postnatal weeks (Butts et al., 2014). PCs, on the other hand, form a single lamina at the early postnatal stage, and develop their spectacular dendritic trees thereafter (Kapfhammer, 2004). All these processes are known to be regulated by various hormonal factors such as thyroid hormone (Koibuchi, 2013) and estradiol (Haraguchi et al., 2012). It is worth noting that certain of gastrointestinal (GI) peptides, including vasoactive intestinal polypeptide (VIP) and pituitary adenylate cyclase-activation polypeptide (PACAP), are constitutively expressed in cerebellar cortex (Joo et al., 2004). Moreover, PACAP has been found to regulate GC proliferation and migration, and to suppress cell apoptosis (Vaudry et al., 2000). Interestingly, these peptides share sequence homology with secretin (SCT) and cross-reactivity with secretin receptor (SCTR; Laburthe and Couvineau, 2002). Taken together, it can be speculated that SCT may share similar functions in mediating cerebellar development.

SCT was initially noted to stimulate pancreatic secretion, and later was shown to exert pluripotent roles in regulating various behaviors including feeding, water drinking, and even spatial memory in central nervous system (reviewed in Zhang and Chow, 2014). Our previous study demonstrated the requirement of PC-specific SCT in maintaining intact motor coordination and motor learning function in mice (Zhang et al., 2014). In the cerebellum, SCT and SCTR were constitutively expressed during embryonic and adult stages (Lee et al., 2005; Siu et al., 2005), suggesting a potentially developmental role of SCT. As phenotypic evidence, we found that SCT deprivation retarded the onset of motor reflexes during the first two postnatal weeks (Zhang et al., 2014). Moreover, it was shown that SCT or SCTR deficiency enhanced the susceptibility of neural progenitor cells against apoptosis (Hwang et al., 2009; Jukkola et al., 2011). SCT can also induce neurite outgrowth of cultured neurons (Kim et al., 2006). However, there has been no *in vivo* study that directly describe the effect of SCT on postnatal development of the cerebellum.

In the present study, we found prominent and constitutive expression of SCT and SCTR in cerebellar cortex during the postnatal period. Using SCT knockout (Sct−/−) mice, we provided direct evidence indicating that SCT deprivation primarily altered the developmental patterns of PCs and GCs. Striking features included the remarkably decreased PC density and dendritic complexity, in addition to an apparently thinner EGL in Sct−/− mice. The latter was found to result from accelerated migration and increased apoptosis of GCs under SCT deprivation. We also provided both *in vivo* and *in vitro* evidences revealing that SCT suppressed apoptosis in the postnatal cerebellum via the cAMP/protein kinase A (PKA) and mitogen activated protein kinase (MAPK)/extracellular signal regulated kinase (ERK) signaling pathways. Collectively, our results established previously unrecognized functions of SCT in modulating postnatal development of the cerebellum.

## Materials and Methods

### Animals

Sct−/− mice were generated in our lab as previously described (Lee et al., 2010) and have been backcrossed with C57BL/6J mice for at least 10 generations (*N* ≥ 10). All mice were kept in a temperature-controlled animal facility with a 12/12-h normal light/dark cycle and were fed with food and water *ad libitum*. The genotype of offspring was determined by PCR analysis with the genomic DNA prepared by Terra™ PCR Direct Polymerase Mix (Clontech Laboratories, Mountain View, CA, USA) following the user manual. At least two different litters containing both Sct−/− and Sct+/+ mice were used in each experiment and all experiments were performed blind to the genotype of the mice. All animals experiment protocols were approved by Committee on the Use of Living Animals in Teaching and Research of the University of Hong Kong.

### Quantitative Real-Time PCR

Total RNA was extracted from the cerebellum using Trizol (Invitrogen, Carlsbad, CA, USA) and reversely transcribed into cDNA using Transcriptor First Strand cDNA Synthesis Kit (Roche, Mannheim, Germany). Quantitative real-time PCR was performed using SYBR PCR Master Mix kit (Applied Biosystems, Foster City, CA, USA) on StepOnePlus™ real-time PCR system (Applied Biosystems). The primer sequences were: Sct, forward 5′-GACCAT GGAGC CTCCG CTG-3′, Reverse 5′-GGGAC AGCCT GGGCA GAAGC C-3′; Sctr, forward 5′-CAGGC TGCAA GCTGG TCATG-3′, reverse 5′-CCAGA AAGTG TCTGG TGACA G-3′; Gapdh, forward 5′-TGTGT CCGTC GTGGA TCTGA-3′, reverse 5′-CCTGC TTCAC CACCT TCTTG AT-3′. Relative mRNA levels of target genes were normalized to Gapdh housekeeping gene and determined by the 2^−∆∆Ct^ method (Livak and Schmittgen, 2001). Average values were taken from three to four animals at each time point for statistical analysis.

### Immunofluorescent Staining

8-μm sagittal sections prepared from the formalin-fixed paraffin-embedded cerebellum were deparaffinized in xylene and rehydrated in gradient ethanol. Antigen retrieval was performed by microwave heating in 10 mM sodium citrate buffer. Non-specific binding sites were blocked by 5% BSA for 1 h at room temperature. The sections were incubated with primary antibody (1:500) against SCT (Abmart, Shanghai, China), SCTR (Abmart, Shanghai, China), or Calbindin D-28K (Abcam, Cambridge, UK) overnight at 4°C and then Alexa Fluor 488 donkey anti-rabbit secondary antibody (1:500; Invitrogen) for 1 h at room temperature. The sections were counterstained with 0.5 μg/mL Hoechst 33258 (Invitrogen). Images were captured using a Nikon 80i fluorescent microscope (Nikon, Tokyo, Japan).

### Cerebellar Morphometry

The linear PC density was calculated by dividing the total number of PCs by the length of PC layer along the lobule IV/V. The EGL thickness was measured at six sites along the lobule IV/V on the same sections. The length of PC monolayer and the EGL thickness was quantified using SPOT Advanced (SPOT Imaging, Sterling Heights, MI, USA). The morphometric measurements were performed on 8–10 consecutive sections per animal. Average values were taken from five to six animals of each genotype at each time point for statistical analysis.

Golgi staining of individual PC was performed using FD Rapid GolgiStain™ Kit (FD NeuroTechnologies, Inc., Ellicott City, MD, USA) according to the manual instruction. PCs close to the apical regions of cerebellar folia were captured under a bright-filed microscope (Zeiss, Germany). The soma and dendrites of PCs were plotted and analyzed using NeuroLucida software (MBF Bioscience, Willison, VT, USA). Sholl analysis was performed, in which the soma was localized as the center of concentric circles with 10-μm intervals. The number of intersections of dendritic branches and the total length of dendrites within each circle were calculated. To evaluate dendritic spine density, the numbers of spines from both proximal and distal dendrites were counted and the spine density was calculated by dividing the number of spines by the dendritic length.

### *In Vivo* Migration Assay

A single dose of EdU (50 mg/kg, Click-iT^®^ EdU Alexa Fluor^®^ 594 Imaging Kit, Invitrogen) was intraperitoneally injected into P7 mice. The whole brain samples were harvested at different time points (2, 24, 48, 72 h) post-injection. Incorporated EdU was detected on 8-μm paraffin-embedded sagittal sections by Click-iT^®^ EdU Alexa Fluor^®^ 594 Imaging Kit following the provided protocol. The number of EdU-labeled cells in the EGL, molecular layer (ML) and internal granular layer (IGL) of each field was determined respectively by ImageJ 1.48 (National Institutes of Health, Bethesda, MD, USA). At least nine fields from three sections were analyzed in each animal. Data from three to five animals of each genotype per time point were used to conduct statistical analysis.

### TUNEL Assay

Terminal deoxynucleotidyl transferase (TdT) dUTP Nick-End Labeling (TUNEL) assay was performed by *in situ* Cell Death Detection Kit, TMR red (Roche) following the manual instruction. After deparaffinization and rehydration, sections were permeabilized with 0.1 M Citrate buffer and incubated with the freshly prepared TUNEL reaction mixture in a dark and humidified chamber for 1 h at 37°C. Nuclei were stained with 0.5 μg/mL Hoechst 33258. The number of TUNEL-positive cells was counted in 9–12 randomly selected fields per animal. Average values were taken from five to six animals of each genotype at each time point for statistical analysis.

### *Ex Vivo* Cerebellar Slice Culture

The preparation of cerebellar slices followed previously reported methods (Hurtado de Mendoza et al., 2011). In brief, cerebella were rapidly dissected from P7 to 10 Sct−/− mice and cut into 300-μm slices using a Vibratome (Campden Instruments Ltd., Loughborough, UK) in the iced artificial cerebrospinal fluid (ACSF) containing 2.5 mM calcium chloride and 10 mM D-Glucose. Cerebellar slices were transferred onto 30-mm culture plate inserts with 0.4-μm pores (Millipore, Billerica, MA, USA) in the 6-well plate containing the culture media (75% MEM, 25% heat-inactivated horse serum, 25 mM HEPES, 1 mM glutamine, 5 mg/mL glucose, 100 U/mL penicillin and streptomycin). To investigate the mechanisms underlying SCT-mediated apoptosis, cerebellar slices were incubated with graded concentration of SCT (0, 0.01, 0.1, 1 and 10 μM; AnaSpec Inc., San Jose, CA, USA) for 30 min. Pretreatment with H89 (20 μM; Sigma-Aldrich, St. Louis, MO, USA), and/or U0126 (20 μM; Cell Signaling Technology, Danvers, MA, USA), or BI-D1870 (20 μM; Santa Cruz Biotechnology, Dallas, TX, USA) for 30 min was performed prior to the 30-min incubation with 1 μM SCT. All slices were incubated in a humidified chamber at 37°C with 5% CO_2_.

### Western Blot

The dissected cerebellum or cultured cerebellar slices were homogenized in ice-cold NP-40 lysis buffer (150 mM sodium chloride, 1.0% NP-40, 50 mM Tris pH 8.0) with freshly prepared protease and phosphatase inhibitors (Roche). Protein lysates with an equal amount were separated by SDS–PAGE and transferred to a nitrocellulose membrane (GE Healthcare Life Sciences, Pittsburgh, PA, USA). The membrane was blocked using 5% defatted milk powder and then incubated with primary antibodies (1:1000) against caspase-3 (Cell Signaling Technology), cleaved caspase-3 (Cell Signaling Technology), phospho-ERK1/2 (Abcam), total-ERK1/2 (Cell Signaling Technology), phospho-p90RSK (Abcam), total-p90RSK (Abcam), phospho-Akt (Abcam), total-Akt (Cell Signaling Technology), phospho-cAMP response element binding (phospho-CREB; Abcam), or total-CREB (Abcam) overnight at 4°C, followed by incubation in HRP-conjugated anti-rabbit IgG antibody (1:5000, Santa Cruz Biotechnology, Dallas, TX, USA) for 1 h at room temperature. Signals were developed with Western Lightning plus-ECL Enhanced Chemiluminescence Substrate (PerkinElmer, Waltham, MA, USA) and the intensity was quantified by ImageJ 1.48 (National Institutes of Health, Bethesda, MD, USA). GAPDH served as the loading control.

### Statistical Analysis

All data are presented as mean ± SEM. All statistical analysis and graph plotting were performed using GraphPad Prism version 6.0 (GraphPad Software, Inc., La Jolla, CA, USA). Data were analyzed using unpaired Student *t*-test, one-way analysis of variance (ANOVA) followed by Tukey’s *post hoc* test or 2-way ANOVA followed by *post hoc* Bonferroni comparison. Significant difference was considered if *p* < 0.05.

## Results

### Characterization of SCT and SCTR Expression in Mouse Postnatal Cerebellum

**Figure 1:**
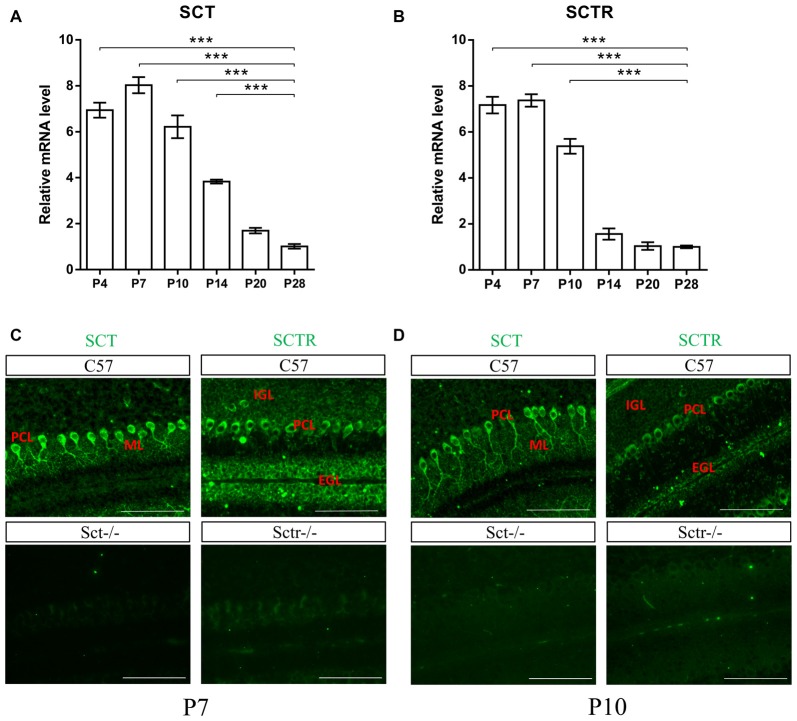
Characterization of secretin (SCT) and secretin receptor (SCTR) expression in mouse postnatal cerebellum. **(A,B)** The transcript levels of SCT **(A)** and SCTR **(B)** in P4, P7, P10, P14, P20 and P28 cerebellar tissue **(C,D)** Immunofluorescent staining of SCT and SCTR on P7 **(C)** and P10 **(D)** cerebellar para-sagittal sections. Sct−/− and Sctr−/− cerebellar sections were included as negative control for SCT and SCTR staining, respectively. Scale bar in **(C,D)**, 100 μm. ***p < 0.001 against the transcript level at P28. *N* = 3–4 animals per group at each time point. Data are shown as mean ± SEM.

To investigate both temporal and spatial expression patterns of SCT and SCTR in postnatal mouse cerebellum, the transcript levels of SCT and SCTR were examined at several critical postnatal stages (P4, P7, P10, P14, P20 and P28). Both SCT and SCTR were significantly and consistently upregulated during the earliest postnatal stages (*p* < 0.001 at P4, P7 and P10 comparing against P28 under one-way ANOVA followed by Tukey *post hoc* test). The expression of SCT and SCTR apparently decreased since P14 but persisted throughout the postnatal period (Figures 1A,B). Using immunofluorescent labeling, no differences in the distribution of SCT and SCTR were found among different cerebellar lobules. We found prominent and almost exclusive SCT expression in the soma and proximal dendrites of PCs while SCTR was expressed in PC soma only. SCTR was also strongly expressed in the immature GCs of EGL during early postnatal period. Sct−/− and Sctr−/− mice showed largely abolished expression of SCT and SCTR, respectively, in the postnatal cerebellum (Figures 1C,D).

### Reduced PC Number and Dendritic Complexity Under SCT Deprivation

**Figure 2:**
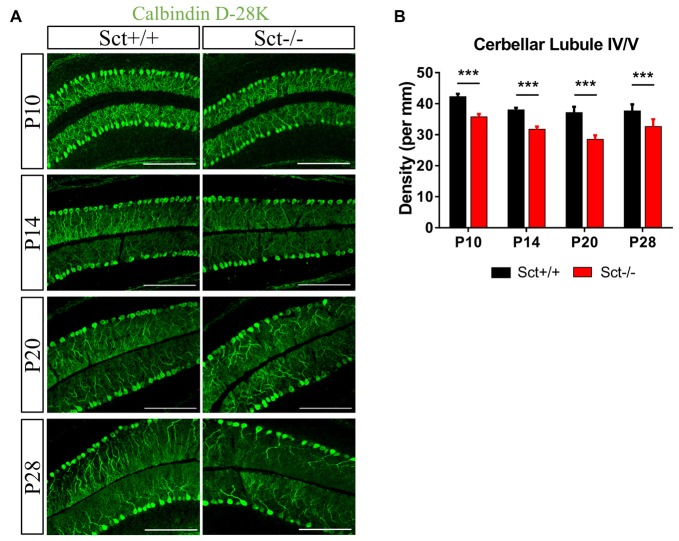
Reduced Purkinje cell (PC) numbers in Sct−/− mice. **(A)** Immunofluorescent staining of Calbindin D-28K on the para-sagittal sections of cerebellar vermis at P10, P14, P20 and P28. **(B)** PC density in the cerebellar lobule IV/V was compared between Sct+/+ and Sct−/− mice. *N* = 5–6 animals per group at each time point. Scale bar in **(A)**, 200 μm. ***p < 0.001. Data are shown as mean ± SEM.

**Figure 3:**
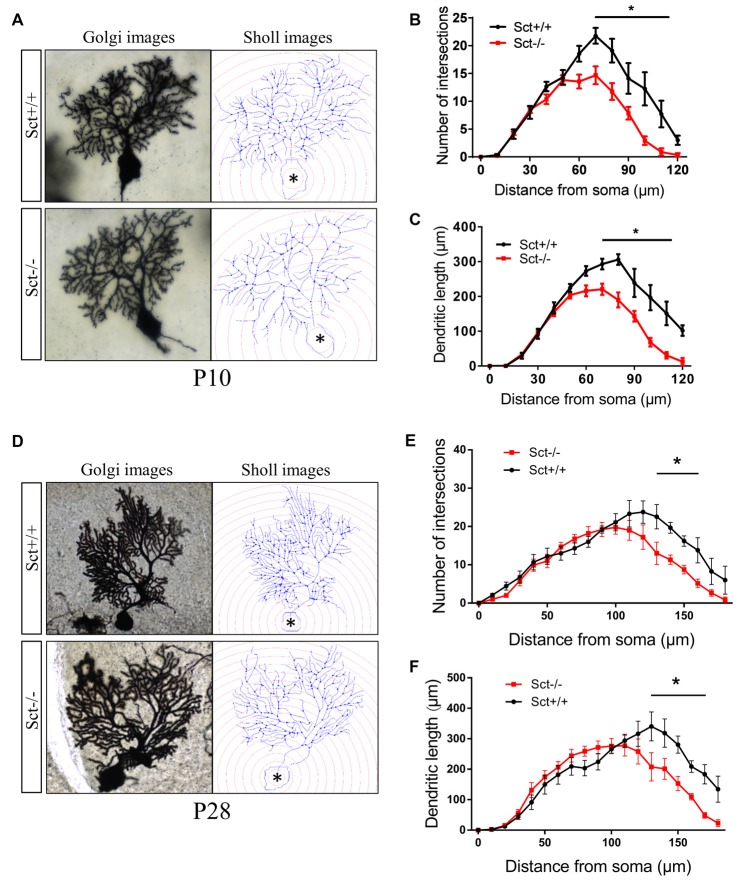
Impaired dendritic arborization of PCs in Sct−/− mice. **(A,D)** Representative photomicrographs of Golgi-stained PCs (left) and the corresponding reconstruction images (right) of PC dendrites at P10 **(A)** and P28 **(D)**, respectively. Concentric circles with a 10-μm interval were plotted from the soma (asterisk). **(B,E)** The number of dendritic branch intersections against arbitrary 10-μm concentric circles at P10 **(B)** and P28 **(E)**. **(C,F)** The total length of dendritic branches within two adjacent concentric circles at P10 **(C)** and P28 **(F)**. *N* = 9–16 cells were reconstructed from three independent animals in each group. *p < 0.05. Data are shown as mean ± SEM.

To explore the role of the constitutive expression of SCT and SCTR in the postnatal cerebellum, we analyzed the cerebellar development under endogenous SCT deprivation. No deficits of the general morphology, foliation pattern or overall size of cerebellum were found in Sct−/− mice (Supplementary Figure S1). However, the density of PCs was significantly decreased in Sct−/− mice since P10 until the adult age P28 (2-way ANOVA with respect to genotype: *F*_(1,37)_ = 213.3, *p* < 0.001; *Post hoc* Bonferroni comparison between Sct+/+ and Sct−/−: *p* < 0.001; Figures 2A,B). To further investigate the dendritic morphology of individual PCs, Golgi staining in conjunction with the Sholl analysis was performed. P10 Sct−/− mice displayed significantly fewer PC dendritic branches and shorter dendritic lengths in the distal (70–110 μm from soma) section (*F*_(1,245)_ = 45.38 or 65.59, *p* < 0.001; Bonferroni comparison: *p* < 0.05; Figures 3A–C). This impaired dendritic arborization persisted in adult P28 mice (Figures 3D–F). More detailed analysis revealed a remarkable decrease in spine density of PC dendrites (*p* < 0.001 by 2-sample student *t*-test; Supplementary Figure S2). These observations suggested dual roles of SCT in PC maintenance and dendritic elaboration.

### SCT Deficiency Accelerates GC Migration

**Figure 4:**
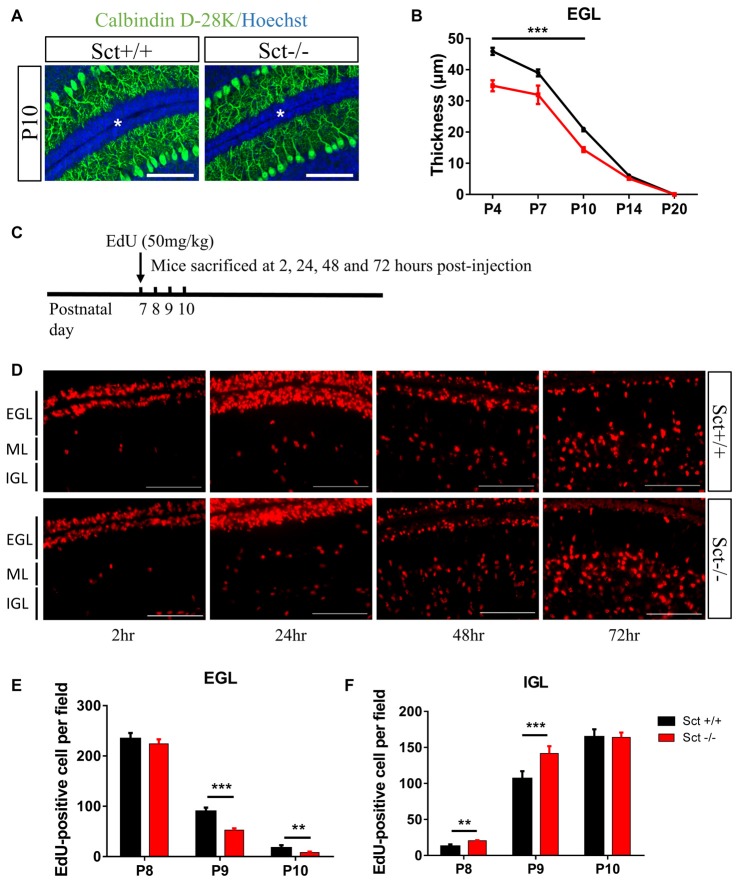
Reduced external granular layer (EGL) thickness and aberrant granular cell (GC) migration in Sct−/− mice.** (A)** Representative images from the P10 Sct+/+ and Sct−/− cerebellar para-sagittal sections double-stained by Calbindin D-28k and nuclear Hoechst. The asterisk indicates the EGL. **(B)** The average thickness of the EGL from P4 to P20. **(C)** Schematic illustration of EdU-labeled cell migration assay. **(D)** EdU staining on the cerebellar para-sagittal sections of Sct+/+ and Sct−/− littermates harvested at the indicated times post-injection. **(E,F)** The average number of EdU-positive cells in the EGL **(E)** and internal granular layer (IGL; **F**) of each field. *N* = 3–5 animals per group. Scale bar in **(A,D)**, 100 μm. ***p < 0.001 and **p < 0.01. Data are shown as mean ± SEM.

Since SCTR was prominently expressed in the EGL (Figures 1C,D), we hypothesized that SCT might also modulate postnatal EGL maturation. Morphometric examination showed that SCT deprivation resulted in a significant decrease of the EGL thickness from P4 to P10 (*p* < 0.001 at P4, P7 and P10 using 2-way ANOVA followed by *post hoc* Bonferroni comparison; Figures 4A,B), while no apparent changes in the thickness of ML and IGL were observed between the two genotypes (Supplementary Figure S3). Postnatal EGL is under dynamic regulation by proliferation and migration processes. Our results showed no significant differences in GC proliferation or the expression of its major driving factor sonic hedgehog (Shh; Lewis et al., 2004) between Sct+/+ and Sct−/− mice (Supplementary Figure S4). We further examined the inward migration of post-mitotic GCs from the EGL by modified EdU labeling assay (Figure 4C), and found more EdU-labeled GCs in the IGL of Sct−/− mice at P8 (*p* < 0.001 using *post hoc* Bonferroni comparison after 2-way ANOVA; Figures 4D,F). The number of EdU-labeled GCs in the EGL was remarkably decreased in Sct−/− mice at P9, accompanied with the consistently higher number of migrating cells in the IGL (*p* < 0.001 using *post hoc* Bonferroni comparison after 2-way ANOVA; Figures 4E,F). On P10, fewer EdU-labeled GCs remained in the EGL of Sct−/− mice (*p* < 0.01 using *post hoc* Bonferroni comparison; Figures 4D,E). Taken together, the initially consistent but later diverged EdU-labeled GC numbers among the EGL and IGL between Sct+/+ and Sct−/− mice indicated accelerated GC migration under SCT deprivation, which, however, did not affect the proliferation.

### Excess Apoptosis in the Cerebellum Under SCT Deprivation

**Figure 5:**
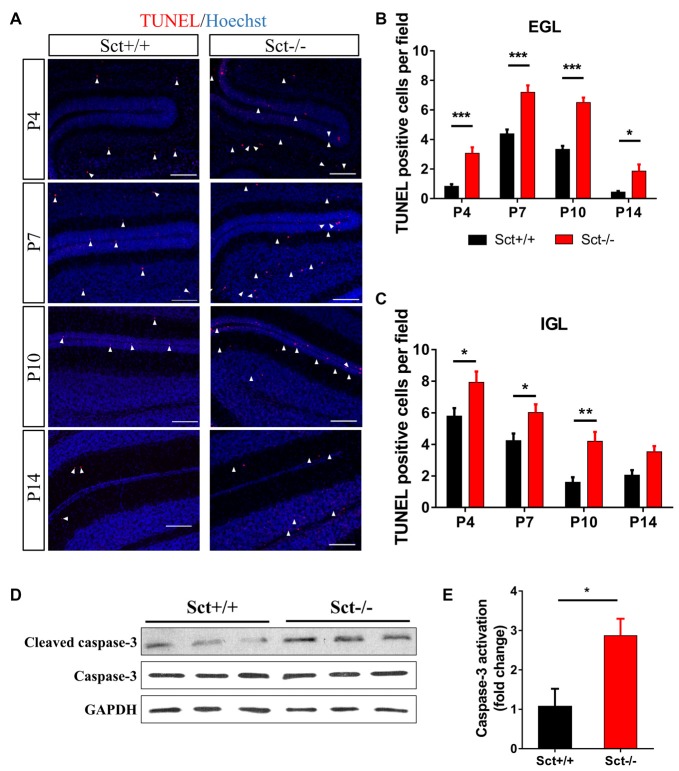
Excess apoptosis during postnatal development in the Sct−/− cerebellum.** (A)** Terminal deoxynucleotidyl transferase (TdT) dUTP Nick-End Labeling (TUNEL) assay and Hoechst counterstaining on the cerebellar para-sagittal sections at P4, P7, P10 and P14. Arrowheads indicate apoptotic cells positive for TUNEL staining (red). **(B,C)** The average number of TUNEL-positive cells in EGL **(B)** and IGL **(C)** of each field at the indicated time points. *N* = 5–6 animals per group. **(D)** Western blot analysis showing the protein expression levels of pro- and cleaved caspase-3 in the P10 cerebellum. GAPDH was included as a loading control. **(E)** The fold change of cleaved (activated) caspase-3 relative to the Sct+/+ control. Scale bar in **(A)**, 100 μm. ***p < 0.001, **p < 0.01 and *p < 0.05. Data are shown as mean ± SEM.

Proliferation and programmed cell death are two critical processes in maintaining GC population during postnatal development. We next investigated if SCT deprivation affected cell apoptosis. *In situ* TUNEL assay revealed significantly more apoptotic cells in the EGL (*Post hoc* Bonferroni comparison: *p* < 0.001; Figures 5A,B) and the IGL (*Post hoc* Bonferroni comparison: *p* < 0.05; Figures 5A,C) from P4 to P14. In line with these observations, the ratio of cleaved caspase 3 against total caspase 3, which can be used to evaluate the apoptotic activity, was also remarkably higher in Sct−/− cerebellum (2-sample student *t*-test, *p* < 0.05; Figures 5D,E). Taken together, SCT knockout significantly elevated apoptosis during postnatal cerebellar development, suggesting a potential effect of SCT on protecting developing neurons from apoptosis.

### Involvement of ERK and PKA Signaling Pathways in SCT-Mediated Neuronal Apoptosis

**Figure 6:**
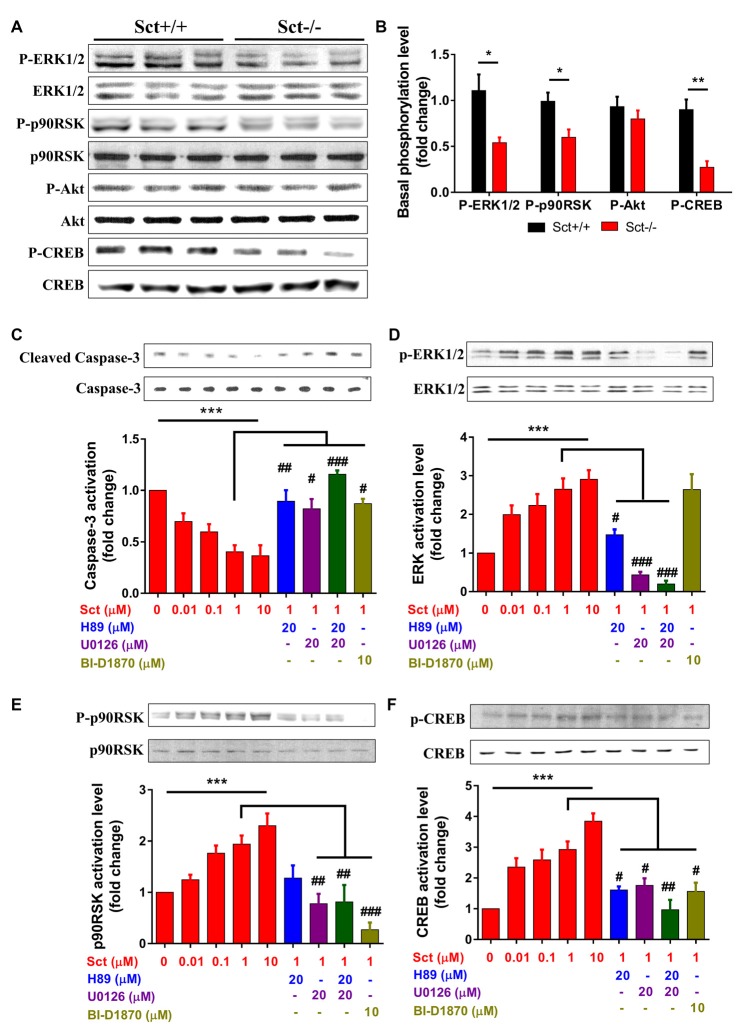
Involvement of MEK/extracellular signalregulated kinase 1/2 (ERK1/2) and cAMP/protein kinase A (PKA) signaling in SCT-inhibited caspase-3 activation SCT-stimulated cAMP response element binding (CREB) phosphorylation.** (A)** The protein expression levels of phosphor (P)-ERK1/2, P-p90RSK, P-Akt and P-CREB in Sct+/+ and Sct−/− littermates. **(B)** The fold change of P-ERK, P90RSK, P-Akt and P-CREB relative to the corresponding Sct+/+ control. **p < 0.01 and *p < 0.05 by 2-sample student *t*-test. **(C–F)** The activation level of caspase-3 **(C)** and the phosphorylation level of ERK1/2 **(D)**, p90RSK **(E)** and CREB **(F)** under the stimulation of graded concentrations of SCT or the incubation of indicated specific inhibitors together with 1 μM SCT. Quantification data were obtained from three to four independent experiments and are shown as mean ± SEM. ***p < 0.001 by one-way analysis of variance (ANOVA); ^###^*p* < 0.001, ^##^*p* < 0.01 and ^#^*p* < 0.05 against 1 μM SCT group using one-way ANOVA followed by Tukey *post hoc* test.

Apoptosis is under orchestrated regulation of multiple signaling pathways, among which phosphatidylinositide 3-kinase (PI3K)/Akt and MAPK/ERK pathways have critical regulating effects (Kennedy et al., 1997; Wada and Penninger, 2004). In addition, activation of the CREB protein, which is the common downstream target effector of PI3K/Akt, MAPK/ERK and cAMP/PKA pathways, can promote cell survival and inhibit apoptosis (Shaywitz and Greenberg, 1999; Finkbeiner, 2000). To elucidate the mechanisms by which SCT regulates apoptosis, activity of these critical signaling molecules was examined by Western blot. The phosphorylation levels of ERK1/2 and its downstream effector p90RSK were significantly attenuated by the deprivation of SCT (*p* < 0.05 by 2-sample student *t*-test in both cases), although the level of phosphorylated Akt remained unchanged (Figures 6A,B). In addition, SCT deficiency also decreased the basal phosphorylation level of CREB (*p* < 0.01 by 2-sample student *t*-test; Figures 6A,B).

**Figure 7:**
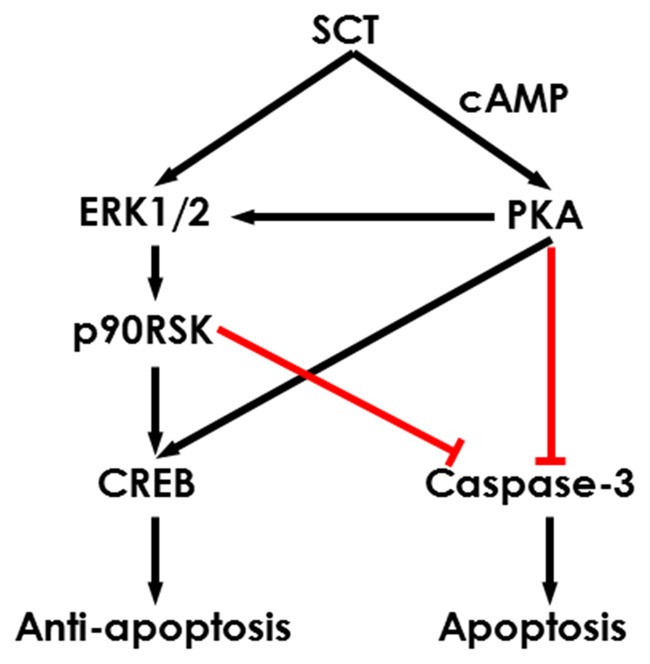
Schematic diagram showing the potential intracellular signaling pathways in the SCT-inhibited neuronal apoptosis. SCT can induce CREB phosphorylation and inhibit caspase-3 activation via mitogen activated protein kinase (MAPK)/ERK1/2 and cAMP/PKA signaling to prevent cerebellar apoptosis.

To further characterize the signaling pathways involved in SCT-mediated apoptosis, pharmaceutical treatments were performed on *ex vivo* cultured para-sagittal cerebellar slices from Sct−/− mice. We found that caspase-3 activation was negatively correlated with SCT dosage, and this inhibitory effect reached saturation at above 1 μM (one-way ANOVA, *p* < 0.001; Figure 6C). The addition of PKA inhibitor H89, and/or ERK1/2 inhibitor U0126, or p90RSK inhibitor BI-D1870 all remarkably abolished the anti-apoptotic effect of SCT, as proved by elevated caspase-3 cleavage (Tukey *post hoc* test against 1 μM SCT, *p* < 0.05; Figure 6C). Notably, the anti-apoptotic effect of SCT can only be fully abolished by co-treatment of H89 and U0126 (Figure 6C). Moreover, ERK1/2 and its downstream effector p90RSK were partially suppressed in the presence H89 (Tukey *post hoc* test, *p* < 0.05; Figures 6D,E). In a similar manner, CREB phosphorylation was restored by SCT, and this recovery was partially suppressed by H89 or U0126, and fully suppressed by adding both drugs together (Figure 6F), indicating the convergence of PKA and ERK signaling pathways towards CREB. In summary, both *in vivo* and *ex vivo* data suggested that SCT prevented cerebellar apoptosis by activating ERK1/2 and PKA pathways in a synergistic manner (Figure 7).

## Discussion

Well-orchestrated regulation of postnatal development is of critical importance for cerebellar function, as disruption of those processes is correlated with neurodevelopmental disorders such as autism spectrum disorder (ASD; Fatemi et al., 2012; Wang et al., 2014) and Joubert syndrome and related disorders (JSRDs; Joubert et al., 1969; Romani et al., 2013). In the present study, we obtained the first piece of *in vivo* evidence showing the importance of SCT in cerebellar postnatal development as SCT deprivation resulted in loss of PCs, irreversible PC dendritic abnormality, and a thinner EGL by inducing accelerated migration and excess apoptosis of GCs. Further mechanistic studies for the first time revealed the involvement of cAMP/PKA and MAPK/ERK signaling pathways in SCT-mediated anti-apoptosis.

Both SCT and SCTR have been found in cerebellar primordium (Siu et al., 2005, 2006) and in adult cerebellum (Yung et al., 2001; Lee et al., 2005). Our results demonstrated the sustained expression of SCT and SCTR in the postnatal cerebellum. These findings collectively revealed the constitutive expression of SCT/SCTR axis in the cerebellum from embryonic to adult stage. Of note, SCT and SCTR are abundantly expressed during the first two postnatal weeks, when PCs and GCs are undergoing rapid development. We also showed the presence of SCTR in postnatal PCs and GCs, strongly indicating the role of SCT in their postnatal development. In the present study, we firstly found decreased PC numbers in Sct−/− mice since P10 until adult P28 in the cerebellar lobule IV/V. The anterior lobules I–V are known to preferentially regulate the body sensorimotor functions (Fatemi et al., 2012). Our histological observation of the lobule IV/V is thus in line with previous work showing impaired motor coordination function in Sct−/− mice (Zhang et al., 2014). It is worth noting that the PC maintenance appears to arise from a cell-autonomous effect of SCT, as a comparable reduction of PC density was found in Purkinje-specific SCT knockout (Pur-Sct−/−) mice (Supplementary Figure S5). SCT may bind to SCTR on PCs in an autocrine manner to regulate PC development. However, whether the decreased PC number is due to impaired primary proliferation, or secondary apoptosis remains unclear at the current stage. The dendritic arborization of PCs occurs during the first three postnatal weeks in mice (Leto et al., 2016), and is governed by both intrinsic and extrinsic factors (Sotelo and Dusart, 2009). We found a persistent impairment of PC dendritic formation under SCT deficiency. This is in agreement with the defective dendritic spines of hippocampal CA1 pyramidal neurons under SCTR deprivation (Nishijima et al., 2006), while in contrast with a later study showing SCT deprivation did not cause any significant change of dendritic branches (Yamagata et al., 2008). Thus, PCs may have higher vulnerability toward SCT deficiency comparing to CA1 region. An *in vitro* study showed that SCT facilitated dendritic growth via PKA-MAPK pathway (Kim et al., 2006). CREB also plays a critical role in mediating dendritic growth through activation of a specific transcriptional program (Redmond et al., 2002; Wayman et al., 2006; Sargin et al., 2013). Since our study demonstrated SCT-induced activation of the PKA-MAPK and PKA-CREB pathways in *ex vivo* cerebellar slices, future studies will determine whether SCT regulates *in vivo* PC dendritic arborization through these two pathways. As intact PC dendritic arborization and wiring with presynaptic neurons are indispensable for synaptic transmission, further studies can be pursued to examine synaptic formation and transmission in Sct−/− mice as evidence for behavioral deficits of these animals (Zhang et al., 2014).

In Sct−/− mice, EGL showed a decreased thickness. Several possible explanations for this finding were explored, including decreased proliferation of GC progenitors, accelerated migration of post-mitotic GCs and increased GC apoptosis. The first possibility that the thinner EGL under SCT deprivation could be associated with the disrupted GC proliferation was ruled out as similar proliferation activity existed in Sct−/− cerebellum. Nevertheless, accelerated GC migration was substantiated by the early arrival of GCs to the IGL in Sct−/− mice. Various intracellular molecules govern GC migration. It is well known that SCT mainly activates the cAMP-dependent pathway in a number of brain regions such as cerebellum, amygdala and hypothalamus (Yung et al., 2001; Chu et al., 2009; Pang et al., 2015). Notably, cAMP can endow a “pause” signal on migrating GCs (Kumada et al., 2006) and it has been demonstrated that PACAP can retard GC migration by activating the cAMP/PKA signaling pathway (Cameron et al., 2007). Likewise, it is highly possible that SCT retards GC migration from the EGL at least partially through cAMP signaling pathways.

Pathological conditions such as ethanol toxicity can induce significant apoptosis of GCs (Oliveira et al., 2014). It has been reported that SCT signaling is required for the survival of neuronal progenitors under normal and toxic conditions (Hwang et al., 2009; Jukkola et al., 2011). As a consequence of the early arrival to the IGL, the inability of immature GCs to establish proper synaptic connections with target neurons results in cell death (Raff et al., 1989). In consistent with these studies, excess apoptosis of GCs was found in both EGL and IGL of Sct−/− mice. Furthermore, the anti-apoptotic effect of SCT was directly demonstrated as SCT treatment suppressed caspase-3 activation in a dose-dependent manner. Both PKA and ERK1/2-p90RSK signaling are known to suppress caspase-3 activation (Parvathenani et al., 1998; Wada and Penninger, 2004) and accordingly, we found that SCT-induced inhibition of caspase-3 was largely abolished by specific PKA, ERK1/2 or p90RSK inhibitors. It is noteworthy that SCT-induced activation of ERK1/2-p90RSK pathway and suppression of caspase-3 were only partially dependent on PKA stimulation, suggesting a PKA-independent regulatory pathway by SCT through the ERK signaling cascade. Activation of PKA and ERK1/2-p90RSK has also been identified to upregulate CREB (Shaywitz and Greenberg, 1999), which has neuroprotective effects via transcriptional regulation (Jeong et al., 2011). In a similar manner, we found that SCT activated CREB through the PKA and ERK1/2 pathways. In summary, SCT-triggered ERK1/2 and PKA signaling pathways converge on caspase-3 and CREB, by which SCT exerts the neuroprotective role.

ASD is a neurodevelopmental disorder characterized by impairments of social and learning skills and repetitive behavior. Interestingly, SCT or SCTR deficiency results in autistic-like traits, including impaired social recognition (Nishijima et al., 2006; Takayanagi et al., 2017) and motor learning (Jukkola et al., 2011; Williams et al., 2012; Zhang et al., 2014), while SCT treatment attenuates autistic-like behaviors such as repetitive movements and hyperactivity (Köves et al., 2011; Heinzlmann et al., 2012). Furthermore, some of the cerebellar deficits in Sct−/− mice are in line with previous studies on both ASD patients and rodent models, which reported decreased cerebellar PC number (Fatemi et al., 2012; Skefos et al., 2014), impaired PC dendritic formation (Peter et al., 2016), and GC apoptosis (Yochum et al., 2008). Taken together, these studies suggest a possible involvement of SCT in cerebellar pathology of ASD. More substantiated proof can be obtained on Pur-Sct−/− mice. In summary, our current work reveals a previously unrecognized role of SCT in postnatal development of the cerebellum. A role of the cAMP/PKA and MAPK/ERK signaling pathways in SCT-mediated anti-apoptosis has also been established for the first time.

## Author Contributions

LW conducted the experiments and analyzed the results. LW and LZ drafted the manuscript. LZ and BKCC supervised the project. All authors reviewed the manuscript.

## Conflict of Interest Statement

The authors declare that the research was conducted in the absence of any commercial or financial relationships that could be construed as a potential conflict of interest.
