# Supplementary material for: Secretin Modulates the Postnatal Development of Mouse Cerebellar Cortex Via PKA- and ERK-dependent Pathways
Source: Front Cell Neurosci. 2017 Nov 30;11:382. doi: 10.3389/fncel.2017.00382 (PMC5714926; doi:10.3389/fncel.2017.00382)
Supplement: Supplementary file 1 [file Presentation_1.pdf]

## *Supplementary Material*

# Secretin modulates the postnatal development of mouse cerebellum via PKA- and ERK-dependent pathways

**Lei Wang[1], Li Zhang[2] and Billy K C Chow[1]***

*** Correspondence:** Billy Kwok Chong Chow bkcc@hku.hk

## Supplementary Figures

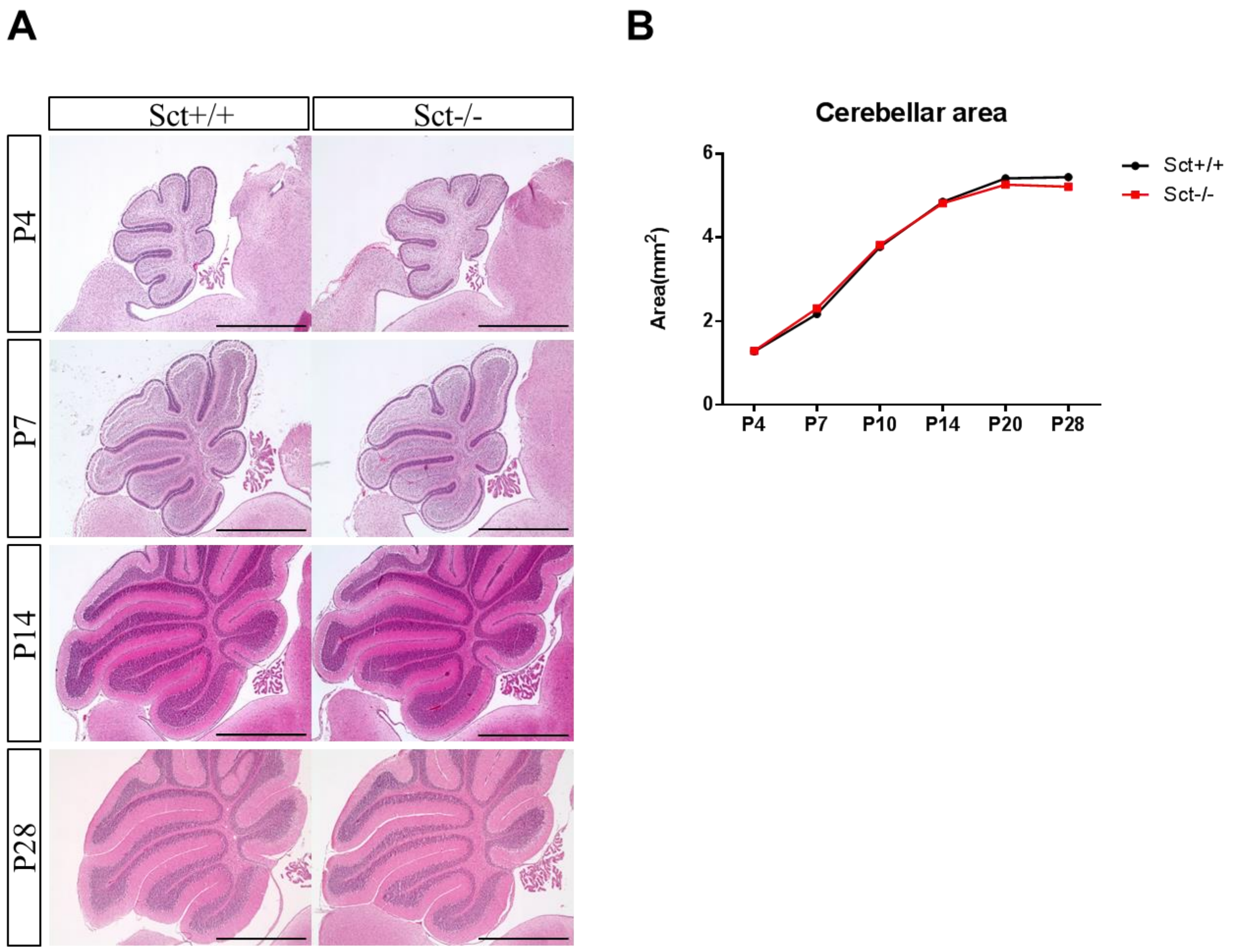


**Supplementary Figure 1. General morphology of cerebellum during postnatal development.** (A) HE staining on the cerebellar para-sagittal sections from Sct+/+ and Sct-/- littermates at P4, P7, P14, and P28. (B) The comparison of the cerebellar area between the two genotype groups at the indicated time points. Scale bar in (A), 1000μm. Data are shown as mean±SEM.

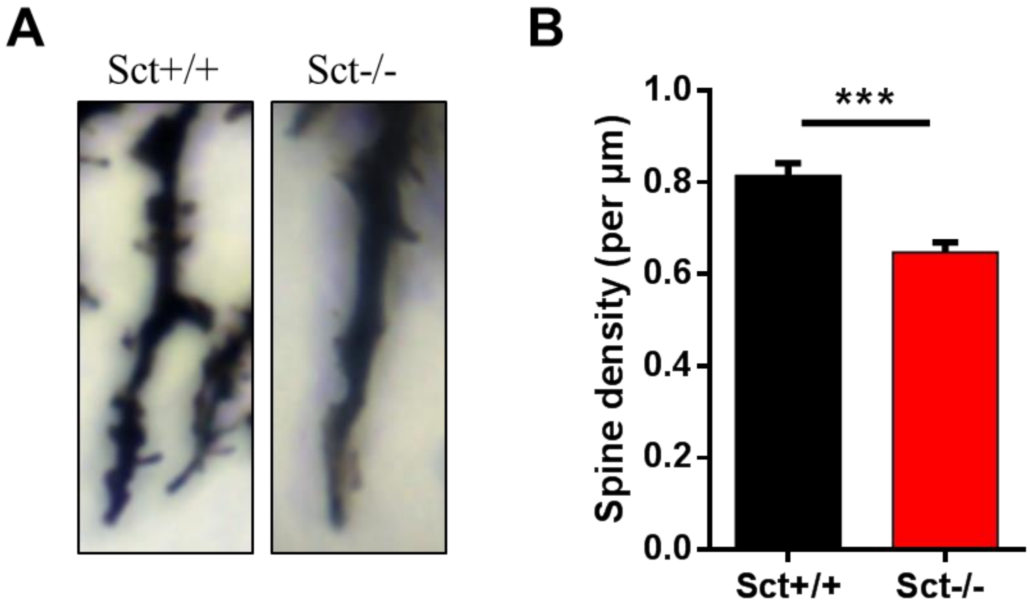


**Supplementary Figure 2.** Reduced spine density of PC dendrites in Sct-/- mice. (A) Higher magnification images of PC dendrites at P10. (B) Quantification of dendritic spine density. N=9~16 cells were reconstructed from 3 independent animals in each group. *** $p<0.001$. Data are shown as mean ±SEM.

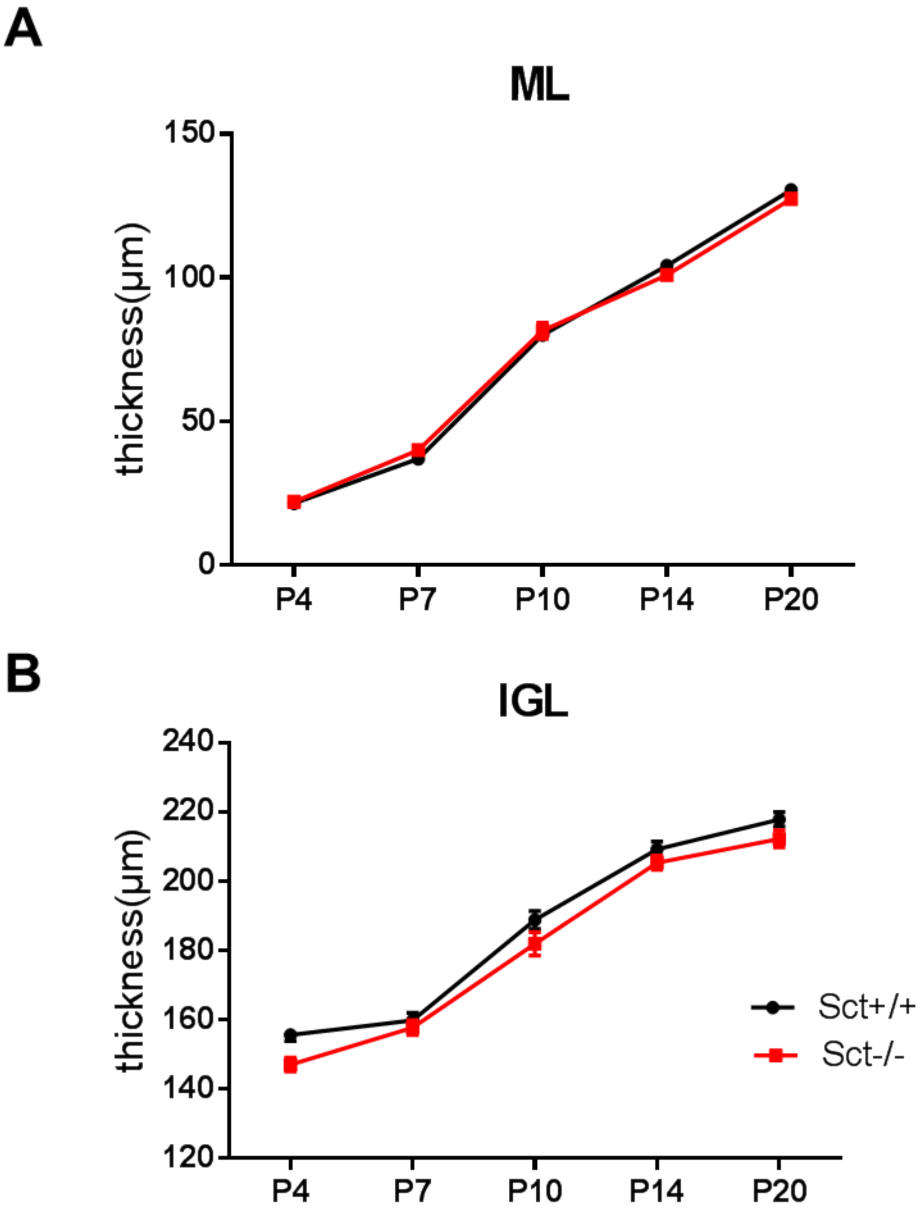


**Supplementary Figure 3. Similar thickness of ML and IGL between Sct+/+ and Sct-/- mice.** Morphometry of the ML (A) and IGL (B) was performed at P4, P7, P10, P14, and P20. N=3~5 animals per group at each time point. Data are shown as mean ±SEM.

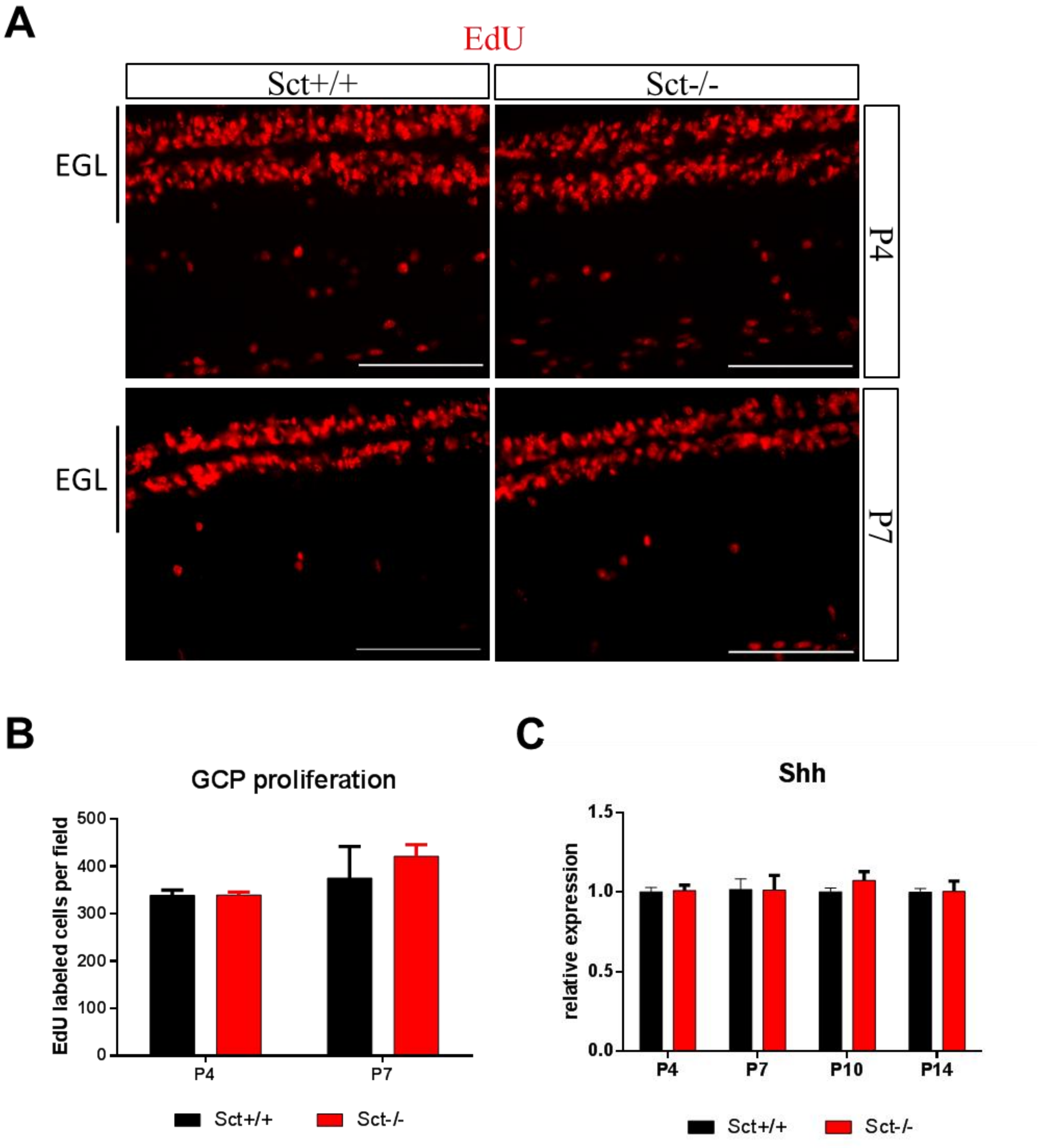


**Supplementary Figure 4. Unimpaired proliferation pattern of granular cells in Sct-/- mice.** (A) EdU staining on the cerebellar para-sagittal sections of Sct+/+ and Sct-/- littermates harvested at 2 hours post-injection. (B) The average number of EdU-positive cells of each field. N=3~5 animals per group. (C) The transcript levels of Shh in P4, P7, P10, and P14 cerebellar tissue. Scale bar in (A), 100μm. N=5~6 per group at each time point. Scale bar in (A), 100μm. Data are shown as mean ±SEM.

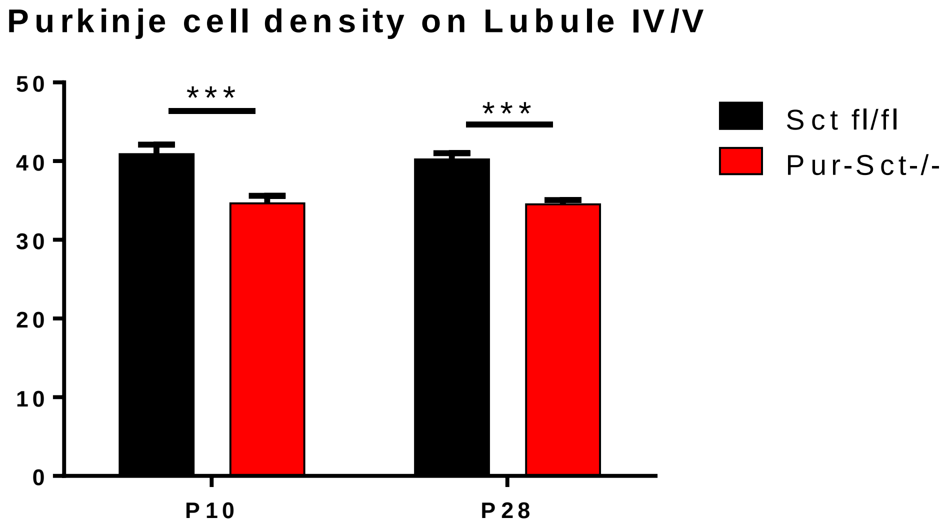


**Supplementary Figure 5. Comparable reduction of PC density under Purkinje-specific SCT deprivation.** Shown is the PC density in the cerebellar lobule IV/V at P10 and P28. N=5~6 animals per group at each time point. ***, $p<0.001$. Data are shown as mean ±SEM.
